# The human fetal adrenal produces cortisol but no detectable aldosterone throughout the second trimester

**DOI:** 10.1186/s12916-018-1009-7

**Published:** 2018-02-12

**Authors:** Zoe C. Johnston, Michelle Bellingham, Panagiotis Filis, Ugo Soffientini, Denise Hough, Siladitya Bhattacharya, Marc Simard, Geoffrey L. Hammond, Peter King, Peter J. O’Shaughnessy, Paul A. Fowler

**Affiliations:** 1 Institute of Biodiversity, Animal Health & Comparative Medicine, College of Medical, Veterinary & Life Sciences, University of Glasgow, Glasgow, G61 1QH UK; 2 Institute of Medical Sciences, School of Medicine, Medical Sciences & Nutrition, University of Aberdeen, Foresterhill, Aberdeen, AB25 2ZD UK; 3 Institute of Applied Health Sciences, School of Medicine, Medical Sciences & Nutrition, University of Aberdeen, Foresterhill, Aberdeen, AB25 2ZD UK; 4 Department of Cellular and Physiological Sciences, Life Sciences Institute, University of British Columbia, British Columbia, V6T 1Z3 Canada; 5 Centre for Endocrinology, William Harvey Research Institute, Barts and the London, Queen Mary University of London, Charterhouse Square, London, EC1M 6BQ UK

**Keywords:** Human, Adrenal, Fetus, Steroid, Maternal smoking

## Abstract

**Background:**

Human fetal adrenal glands are highly active and, with the placenta, regulate circulating progesterone, estrogen and corticosteroids in the fetus. At birth the adrenals are essential for neonate salt retention through secretion of aldosterone, while adequate glucocorticoids are required to prevent adrenal insufficiency. The objective of this study was to carry out the first comprehensive analysis of adrenal steroid levels and steroidogenic enzyme expression in normal second trimester human fetuses.

**Methods:**

This was an observational study of steroids, messenger RNA transcripts and proteins in adrenals from up to 109 second trimester fetuses (11 weeks to 21 weeks) at the Universities of Aberdeen and Glasgow. The study design was balanced to show effects of maternal smoking.

**Results:**

Concentrations of 19 intra-adrenal steroids were quantified using liquid chromatography and mass spectrometry. Pregnenolone was the most abundant steroid while levels of 17α-hydroxyprogesterone, dehydroepiandrosterone sulphate (DHEAS) and progesterone were also high. Cortisol was present in all adrenals, but aldosterone was undetected and Δ^4^ androgens were low/undetected. *CYP17A1*, *CYP21A2* and *CYP11A1* were all highly expressed and the proteins localized to the adrenal fetal zone. There was low-level expression of *HSD3B* and *CYP11B2,* with HSD3B located mainly in the definitive zone. Maternal smoking altered fetal plasma adrenocorticotropic hormone (ACTH) (*P* = 0.052) and intra-adrenal progesterone, 17α-hydroxyprogesterone and 16α-hydroxyprogesterone, but not plasma or intra-adrenal cortisol, or intra-adrenal DHEAS. Fetal adrenal *GATA6* and *NR5A1* were increased by maternal smoking.

**Conclusions:**

The human fetal adrenal gland produces cortisol but very low levels of Δ^4^ androgens and no detectable aldosterone throughout the second trimester. The presence of cortisol in fetal adrenals suggests that adrenal regulation of circulating fetal ACTH remains a factor in development of congenital adrenal hyperplasia during the second trimester, while a relative lack of aldosterone explains the salt-wasting disorders frequently seen in extreme pre-term neonates. Finally, maternal smoking may alter fetal adrenal sensitivity to ACTH, which could have knock-on effects on post-natal health.

**Electronic supplementary material:**

The online version of this article (10.1186/s12916-018-1009-7) contains supplementary material, which is available to authorized users.

## Background

The human fetal adrenal gland develops initially as part of the adrenogonadal primordium and is distinct in the human by 7–8 weeks of gestation. The adrenals secrete cortisol in response to adrenocorticotropic hormone (ACTH) as early as week 8 of gestation [1], although the main steroids produced in fetal life are dehydroepiandrosterone (DHEA) and its sulphate (DHEAS), which act as substrates for placental estrogen production [2]. Together, the fetal adrenal glands and placenta dominate human fetal steroid endocrinology in a manner seen only in higher primates. Normal development and function of the fetal adrenals is also essential for several processes that can affect the fetus itself or the health of the neonate. For example, disruption of the fetal adrenals can lead to disorders of sex development [3], while fetal misprogramming of the stress axis, through altered fetal cortisol secretion, may predispose to diseases in later life [4]. The adrenal glands are also essential for survival early in post-natal life through the secretion of aldosterone, which prevents salt-wasting disorders [5], while adequate cortisol is required to prevent adrenal insufficiency in the newborn [6]. Interestingly, pre-term neonates are prone to salt-wasting disorders, which may indicate a failure of aldosterone synthesis in fetal life or altered sensitivity to the steroid [5]. Despite the importance of the adrenal glands for fetal and post-natal health, however, their development during fetal life in the human is not well described or understood. Animal models are of limited relevance due to significant species differences in fetal adrenal structure, steroidogenic pathways and endocrinology of pregnancy. However, only a limited number of studies have examined the human fetal adrenal directly ([1, 7–11] and in review [12, 13]). Consequently, we have examined human fetal adrenal steroidogenesis during the second trimester in a large number of well-documented, normal, human fetuses. This includes the first comprehensive liquid chromatography (LC)/mass spectrometry (MS) analysis of human fetal adrenal steroid levels during the second trimester.

Maternal smoking during pregnancy remains a significant public health issue, as it can disrupt normal fetal programming and can have irreversible effects on the post-natal life of the offspring [14]. Cigarette smoke contains many potential toxicants (e.g. heavy metals, aldehydes, nitrosamines and polycyclic aromatic hydrocarbons) [15] that can cross the placental barrier to reach the fetus. However, mechanisms behind the long-term effects of smoking on the human fetus remain largely unknown. Maternal smoking can affect fetal adrenal function and development of the hypothalamo-pituitary-adrenal (HPA) axis [16], which may be one mechanism by which smoking programmes later health deficits of the offspring [17, 18]. We have, therefore, extended our developmental study to examine the effects of maternal smoking on human fetal adrenal steroidogenesis.

## Methods

### Sample collection

The collection of fetal material involved in the adrenal studies was approved by the National Health Service (NHS) Grampian Research Ethics Committees (REC 04/S0802/21). Human fetal kidneys were collected under a separate, newer ethics permission as part of the Scottish Advanced Fetal Research (SAFeR) Study. This was approved by NHS Grampian Research Ethics Committees (REC 15/NS/0123). In all cases, women seeking elective terminations of pregnancy were recruited with full written, informed consent by nurses working independently of the study at the Aberdeen Pregnancy Counselling Service. Maternal data, medications used and self-reported number of cigarettes smoked per day were recorded. Only fetuses from normally progressing pregnancies (determined at ultrasound scan prior to termination) from women over 16 years of age and between 11 and 21 weeks of gestation (7 and 20 weeks for fetal kidneys) were collected following termination by RU486 (mifepristone) treatment (200 mg) and prostaglandin-induced delivery, as detailed previously [19]. Fetuses were transported to the laboratory within 30 min of delivery, weighed, sexed and the crown-rump length recorded. Blood samples were collected by cardiac puncture ex vivo and plasma prepared in heparin-coated tubes was stored at –80 °C. Fetal tissues were snap-frozen in liquid nitrogen and stored at –80 °C or fixed in 10% neutral buffered formalin. Adrenal samples were analysed in four groups: control female, smoke-exposed female, control male and smoke-exposed male, with groups balanced as far as possible for gestational age (Table 1). Maternal smoking status was confirmed by measurement of fetal plasma cotinine using a commercially available kit (Cozart Plc, Abingdon, Kent, UK). Fifty-six fetal kidneys were analysed as a single group and as four groups: control female, smoke-exposed female, control male and smoke-exposed male.

**Table 1:** Morphological data for the mothers and fetuses involved in adrenal studies

Population	Characteristic	Female fetuses		Male fetuses	
		Control	Smoke-exposed	Control	Smoke-exposed
Total adrenal samples	*N*	29	19	31	30
Maternal indices	Age (years)	25.5 ± 1.2	23 ± 1.2	23.8 ± 1.06	26.2 ± 1
	Body mass index (BMI, kg/m^2^)	24.1 ± 0.8	26 ± 1.3	26 ± 0.95	25.8 ± 1.2
	Cigarettes/day	0 ± 0	9.3 ± 0.7	0.2 ± 0.2	10.2 ± 1
Fetal indices	Age (weeks)	14.6 ± 0.4	14.3 ± 0.8	14.6 ± 0.6	14.6 ± 0.5
	Weight (g)	77 ± 13.3	75.4 ± 14.1	83.4 ± 12	91.8 ± 12.9
	Crown-rump length (CRL, mm)	98.7 ± 5.2	95.1 ± 5	101.4 ± 4.3	105.5 ± 5.5
	Ponderal index (weight, g/[CRL, cm^3^])	70.2 ± 6.7	72.8 ± 5.4	68.8 ± 3.3	63.2 ± 1.8
	Plasma cotinine (ng/mL)	6.8 ± 3.3	30.2 ± 5.4	5.3 ± 1.2	41.6 ± 6.4
Sub-population: plasma steroids^a^	*N*	14	16	13	17
Maternal indices	Age (years)	24.3 ± 1.7	23.2 ± 1.3	24.5 ± 1.8	24.7 ± 1.2
	BMI (kg/m^2^)	23.5 ± 1.2	23.8 ± 1.3	26.2 ± 1.4	25.3 ± 1.4
	Cigarettes/day	0 ± 0	11 ± 1.3	0 ± 0	12.5 ± 1.1
Fetal indices	Age (weeks)	15.6 ± 0.6	15.2 ± 0.6	15.2 ± 0.6	15.1 ± 0.6
	Weight (g)	104.8 ± 19.9	93.6 ± 16.7	101.7 ± 22.5	97.1 ± 19.3
	CRL (mm)	109.3 ± 7.7	106.9 ± 5.7	108 ± 7.4	107.2 ± 7.4
	Ponderal index (weight, g/[CRL, cm^3^])	64.9 ± 2.6	64 ± 2.6	73.9 ± 4.7	65 ± 2
	Plasma cotinine (ng/mL)	3.3 ± 1.2	41.6 ± 2.8	2.6 ± 0.9	48.6 ± 2.2
Sub-population: mRNA/protein/steroids^a^	*N*	15	15	15	15
Maternal indices	Age (years)	24.7 ± 1.6	23.2 ± 1.4	22.9 ± 1.3	26.6 ± 1.4
	BMI (kg/m^2^)	24.2 ± 0.8	26.5 ± 1.4	26.23 ± 1.5	24.6 ± 1.6
	Cigarettes/day	0 ± 0	8.8 ± 0.8	0 ± 0	12 ± 1.3
Fetal indices	Age (weeks)	15.3 ± 0.6	15.1 ± 0.6	15.2 ± 1.2	15.2 ± 0.6
	Weight (g)	97 ± 19.5	84.8 ± 17	94.9 ± 18.8	94.7 ± 19.9
	CRL (mm)	106.4 ± 6.8	97.4 ± 6.1	104.3 ± 6.9	102.3 ± 8.7
	Ponderal index (weight, g/[CRL, cm^3^])	66.5 ± 2.3	75.5 ± 6.7	72 ± 4.6	67 ± 3.1
	Plasma cotinine (ng/mL)	7.6 ± 4.6	30.2 ± 5.4	4 ± 1.5	50.9 ± 5.7

^a^Although there is some overlap between the two study sub-populations, not all plasma samples were from fetuses where the adrenal glands were collected. Therefore, the two populations were not analysed together

### Plasma measurements of hormones and binding globulins

Assays were performed using plasma samples from 60 second trimester fetuses. Plasma ACTH levels were measured (25 μL/fetus) using a single Milliplex® MAP Human Pituitary Magnetic Bead Panel 1 kit (ACTH, growth hormone (GH), thyroid-stimulating hormone (TSH), ciliary neurotrophic factor (CNTF), agouti-related protein (AGRP); Millipore Limited, Watford, UK) and analysed using a BioPlex200 system (Bio-Rad Laboratories Ltd, Hemel Hempstead, UK). Intra- and inter-assay coefficients of variation were < 10% and < 15% respectively, sensitivity was 0.91 pg/mL and cross-reactivity was negligible. Cortisol was measured in 50 μL of plasma/fetus using a DetectX® Cortisol Enzyme Immunoassay kit (Arbor Assays, Ann Arbor, MI, USA). Cross-reactivity with other steroids was < 8%, sensitivity was 17.3 pg/mL and inter- and intra-assay coefficients of variation were 5.4% and 8.1% respectively. Corticosteroid-binding globulin (CBG) concentrations were measured by an established ligand-saturation assay [20].

### Protein, DNA and RNA extractions

To avoid sampling error due to heterogeneous morphology of the adrenal gland, only whole human fetal adrenal glands were homogenized in the presence of protease inhibitors (Protease Inhibitor Cocktail, Sigma-Aldrich Company Ltd, Gillingham, UK). Protein, DNA and RNA were extracted from total tissue homogenates with Qiagen AllPrep DNA/RNA/Protein mini kits (Qiagen Ltd, Crawley, UK). RNA was extracted from whole human fetal kidneys in the same way.

### Intra-adrenal steroid quantification with LC/MS

Steroids were extracted from the unused, combined, flow-through fractions of the Qiagen AllPrep DNA/RNA/protein extraction protocol and quantified by LC/MS. This approach was taken to maximize the data gathered from as many fetuses as possible and reflects the unique nature and relative scarcity of the human fetal samples.

#### Steroid extractions

Initially, we determined whether intra-adrenal steroid levels could be accurately measured from the unused flow-through fractions of the Qiagen AllPrep DNA/RNA/protein extraction protocol. The first studies, using a [^3^H]-testosterone recovery standard with mouse adrenal tissue homogenate, showed that 72% of testosterone was recovered in the discardable flow-through fraction from the protein precipitation step (step 15 of the Qiagen manual) with 89.6% total recovery from the total combined waste fractions. In further preliminary studies using LC/MS, the recovery of intra-adrenal steroids (from human fetal adrenals) was measured by comparison of steroid levels in the initial tissue lysate with levels in recovered fractions after RNA/DNA/protein extraction. Steroids were recovered primarily in the protein precipitation step (step 15 in the Qiagen protein/DNA/RNA extraction protocol), with the exception of DHEAS, which was recovered in all fractions. Estimates of steroid recovery efficiencies from combined fractions were as follows: cortisol 87%, androstenedione 122%, 17α-hydroxyprogesterone 113%, 21-deoxycortisol 87%, 11-deoxycortisol 111% and DHEAS 56%. These data show that most steroids could be recovered from tissues with high efficiency during Qiagen extraction. For subsequent studies all of the residue fractions from the Qiagen extraction process (steps 8, 15, 21 and 22) were combined and extracted.

Three deuterated steroids (15 ng) were added as internal standards (ISs) prior to steroid extraction, as described below. Steroids in the combined residue fractions were extracted twice using ethyl acetate (4:1 ratio to sample volume), subjected to a solid-phase extraction using 1 mL Strata-X, 33 μm polymeric reverse phase cartridges (Phenomenex, Torrance, CA, USA) and dissolved in a final volume of 150 μL methanol. The percentage recovery of all steroids through ethyl acetate and solid phase extractions was > 82%, and the percent relative standard deviation (RSD; *n* = 6) of all steroid measurements was between 1.82 and 8.75%.

The standards prepared in methanol were not significantly different than those prepared using the extraction matrix, while recovery and percent RSD showed negligible differences. We therefore used standards in methanol for further experiments.

#### Intra-adrenal steroid quantification with LC/MS

Steroids were separated by reversed phase LC on an UltiMate 3000 RSLCnano (Thermo Fisher Scientific, Waltham, MA, USA) separation system, using an Accucore™ PFP LC column with trimethyl silane (TMS) endcapping (50 mm, 2.1 mm, 2.6 μm, Thermo Fisher). For both positive and negative modes, the mobile phase was a mixture of solvents A (1% formic acid in water) and B (49:49:2 methanol:acetonitrile:isopropanol). Steroids were eluted at a flow rate of 0.4 mL/min, using a linear gradient from 24% B to 47% B in 2.0 min, followed by a linear gradient to 66% B in 2.7 min and a subsequent linear gradient to 100% B in 0.1 min. Elution at 100% B was maintained for 0.9 min before lowering it to 24% B over 0.1 min and equilibrating the column for 2.5 min. The total run time was 8.3 min and the injection volume was 5 μL.

Steroids were detected with a Q Exactive Orbitrap (Thermo Fisher) mass spectrometer, using full-scan detection (250–500 m/z). Table 2 contains a summary of the limits of quantification for all steroids in this study. For each sample, one MS run was completed at full scan in positive mode and another MS run at full scan in negative mode. MS mode was found to be more suited for steroid profiling with the Orbitrap MS system, and the sensitivities were similar to those achieved using LC-MS/MS with a triple quadrupole MS system [21].

**Table 2:** Intra-adrenal steroids measured by LC/MS

			Total		Control female		Smoke-exposed female		Control male		Smoke-exposed male	
Steroid	LOQ (ng/mL MeOH)	LOQ (adjusted)^a^	QS^b^ (*n* = 60)	Mean ± SEM (ng/mg tissue)	QS (*n* = 15)	Mean ± SEM (ng/mg tissue)	QS (*n* = 15)	Mean ± SEM (ng/mg tissue)	QS (*n* = 15)	Mean ± SEM (ng/mg tissue)	QS (*n* = 15)	Mean ± SEM (ng/mg tissue)
Pregnenolone	10	0.05	58	5.019 ± 0.49	15	4.619 ± 0.79	13	4.299 ± 0.927	15	4.073 ± 0.828	15	7.087 ± 1.135
17α-Hydroxyprogesterone	1	0.005	60	1.291 ± 0.182	15	1.086 ± 0.151	15	1.115 ± 0.189	15	0.878 ± 0.159	15	2.087 ± 0.624
DHEAS	0.1	0.0005	60	1.153 ± 0.178	15	1.426 ± 0.412	15	0.782 ± 0.16	15	0.966 ± 0.271	15	1.437 ± 0.468
Progesterone	1	0.005	60	1.089 ± 0.185	15	0.993 ± 0.194	15	0.95 ± 0.278	15	1.066 ± 0.504	15	1.347 ± 0.415
Cortisol	0.1	0.0005	56	0.626 ± 0.087	14	0.713 ± 0.198	14	0.498 ± 0.127	13	0.471 ± 0.142	15	0.824 ± 0.198
Corticosterone	0.1	0.0005	57	0.354 ± 0.075	13	0.265 ± 0.073	14	0.213 ± 0.08	15	0.345 ± 0.153	15	0.593 ± 0.224
16α-Hydroxyprogesterone	0.1	0.0005	59	0.259 ± 0.035	15	0.259 ± 0.036	14	0.18 ± 0.04	15	0.192 ± 0.038	15	0.407 ± 0.116
11-Deoxycortisol	0.1	0.0005	60	0.119 ± 0.017	15	0.148 ± 0.039	15	0.1 ± 0.03	15	0.082 ± 0.02	15	0.146 ± 0.042
Cortisone	1	0.005	37	0.108 ± 0.018	9	0.15 ± 0.047	9	0.096 ± 0.034	9	0.075 ± 0.023	10	0.109 ± 0.037
Testosterone	0.1	0.0005	32	0.032 ± 0.007	9	0.062 ± 0.02	8	0.028 ± 0.013	7	0.017 ± 0.008	8	0.023 ± 0.008
Deoxycorticosterone	0.1	0.0005	39	0.023 ± 0.003	12	0.03 ± 0.006	10	0.018 ± 0.003	10	0.019 ± 0.004	7	0.025 ± 0.009
Corticosterone sulphate	10	0.05	10	0.018 ± 0.01	3	0.022 ± 0.019	2	0.003 ± 0.002	1	0.001 ± 0.001	4	0.045 ± 0.032
11-Dehydrocorticosterone	0.1	0.0005	12	0.003 ± 0.001	4	0.005 ± 0.004	3	0.001 ± 0	2	0.002 ± 0.002	3	0.005 ± 0.003
Aldosterone	1	0.005	0	< LOQ	0	< LOQ	0	< LOQ	0	< LOQ	0	< LOQ
DHEA	10	0.05	0	< LOQ	0	< LOQ	0	< LOQ	0	< LOQ	0	< LOQ
Δ4-Androstenedione	10	0.05	0	< LOQ	0	< LOQ	0	< LOQ	0	< LOQ	0	< LOQ
Δ5-Androstenediol	1	0.005	0	< LOQ	0	< LOQ	0	< LOQ	0	< LOQ	0	< LOQ
17α-Hydroxypregnenolone	100	0.5	0	< LOQ	0	< LOQ	0	< LOQ	0	< LOQ	0	< LOQ
Cortisone sulphate	10	0.05	0	< LOQ	1	< LOQ	1	< LOQ	1	< LOQ	1	< LOQ

^a^Equivalent to ng/mg of tissue LOQ

^b^Quantifiable samples out of *n* samples as stated in brackets

*LOQ* limit of quantitation, *QS* quorum sensing, *SEM* standard error of the mean

All instruments were controlled by Chromeleon software (Thermo Fisher), and data acquisition, peak integration and quantification were performed using Xcalibur 2.2 software (Thermo Fisher). Calibration curves were used to quantify the steroids, using the ratio of the steroid peak area relative to the peak area of a specific deuterated IS that had similar elution time and/or chemical properties. Deuterated cortisol (4-pregnen-11β,17,21-triol-3,20-dione-9,11,12,12-d4; Steraloids, Newport, RI, USA) was used as the IS for aldosterone, cortisol, cortisone, corticosterone, 11-deoxycortisol, cortisone sulphate, corticosterone sulphate, 11-dehydrocorticosterone and Δ5-androstenediol. Deuterated progesterone (4-pregnen-3,20-dione-2,2,4,6,6,17α,21,21,21-d9; Steraloids) was used as the IS for DHEA, 17α-hydroxypregnenolone, 17α-hydroxyprogesterone, 16α-hydroxyprogesterone, testosterone, deoxycorticosterone, progesterone, pregnenolone and Δ4-androstenedione. Deuterated DHEAS (5-androsten-3β-ol-17-one-16,16-d2, sulphate, sodium salt; Steraloids) was used as the IS for DHEAS. Calibration curves were constructed from the LC/MS analyses of six to nine calibrator samples in the range of 1–2000 ng/mL. Calibrator samples contained all three deuterated ISs at a concentration of 100 ng/mL, as well as steroid standards at relevant concentrations from a dilution series in methanol. Steroid standard stocks, from which the calibrator dilution series were made, were all sourced from Sigma-Aldrich or Steraloids.

To determine limits of detection (LODs) and limits of quantification (LOQs), calibrator samples (*n* = 13) were prepared at concentrations ranging from 0.01 ng/mL to 10,000 ng/mL, containing all of the steroids and deuterated ISs at a fixed concentration of 100 ng/mL. These were prepared in both methanol and Qiagen buffer mixture. Calibration curves were generated by performing least-squares regression analysis on peak area ratios relative to the IS at different concentrations, within the sensitivity range of each steroid. The LOD and LOQ were defined as the lowest steroid concentration with a signal-to-noise ratio (S/N) larger than 3 and 10 respectively.

### Real-time reverse transcription polymerase chain reaction (RT-PCR)

Messenger RNA (mRNA) from 60 human fetal adrenals was reversed transcribed using random hexamers and Superscript III (Life Technologies, Paisley, UK) [19, 22, 23]. Real-time RT-PCR was performed using Brilliant II SYBR Green Master Mix (Agilent Technologies, Santa Clara, CA, USA) and an MX3000 cycler (Stratagene, Amsterdam, the Netherlands). mRNA levels were expressed relative to two housekeeping genes (HKGs), *TATA box binding protein* (*TBP*) and *Phosphomannomutase 1* (*PMM1*), which were identified as the optimal HKGs from a total of six HKGs tested using NormFinder [24]. Data are expressed as the geomean of values relative to each HKG multiplied by 10^3^. *NR3C2* transcript levels in human fetal kidneys were measured by real-time PCR using LightCycler 480 SYBR Green I Mastermix (Roche, Basel, Switzerland) and a Roche LightCycler 480 and were expressed relative to *ACTB*. All primers were designed as previously described [22]; the sequences are reported in Table 3. Primers designed to amplify *HSD3B* recognized both type 1 and type 2 isoforms of the enzyme.

**Table 3:** Primers used for RT-qPCR

	Forward	Reverse
*CYP11A1*	tttttgcccctgttggatgca	ccctggcgctccccaaaaat
*CYP11B1*	tgtgtgatgctgccggagga	cgcaatcggttgaagcgcc
*CYP11B2*	aggtggacagcctgcatccctg	gcacatctgggttcagccgc
*CYP17A1*	ccatttcctgaacgcaccgg	agagaggccaaggaaacagggct
*CYP21A2*	cggacctgtccttgggagactactcc	ctgggctctcatgcgctcaca
*DAX1* (*NR0B1*)	cctcccaggtccaagccatcaa	tgagttccccactggagtccct
*GATA6*	aataattccattcccatgactccaacttc	aatacttgagctcgctgttctcggg
*HSD3B* ^a^	ccacaccgcctgtatcattgatgtct	taggagttgggcccggctacct
*MC2R*	tcttccacgcactgcggtacc	catcagcgggaacagcgacg
*MRAP*	cacagacatggccaacgggac	agcaccacgaaggcagccag
*PMM1*	aacatctcgcccatcggcc	tcaaagctgatcatgcctcctcg
*POR*	ttttcagcatgacggacatgattctgt	tttcttcatcttttccacaaagctgctc
*SF-1* (*NR5A1*)	tcccttctgccgcttccagaaat	tgaagccattggcccgaatct
*STAR*	ggctggcatggccacagact	ttgggcagccaccccttga
*SULT2A1*	caagatgtccaattattccctcctgagtgt	tctcgaggaagatctgccatcttctc
*TBP*	aggaaaaaattgaatagtgagacgagttcca	tggactaaagatagggattccgggagt
*NR3C2*	tggcctggatgtggttggatttagg	agaaacttgaccccaccgtctttcc
*ACTB*	ttcctgggcatggagtcctgtg	ttgatcttcattgtgctgggtgcc

^a^Amplifies both *HSD3B1* and *HSD3B2*

### Immunohistochemistry

Adrenal sections (5 μm) were dewaxed and rehydrated, and antigen retrieval was carried out in citrate buffer (PT Module Buffer 1; Thermo Fisher) using a bench top autoclave. Endogenous peroxidase activity was quenched using DAKO Real™ peroxidase block (Agilent Technologies), and 20% Normal Goat Serum (Vector Laboratories, Burlingame, CA, USA) was used to block non-specific binding sites. The primary antibodies were for detection of CYP11A1 (non-commercial rabbit polyclonal antibody (gift from A.H. Payne)), CYP17A1 (non-commercial rabbit polyclonal antibody (gift from I. Mason)), CYP21A2 (polyclonal rabbit antibody (Sigma;HPA048979)) and HSD3B (non-commercial rabbit antibody which recognizes both HSD3B1 and HSD3B2 (gift from I. Mason)). The secondary antibody used was Polyclonal Goat anti-Rabbit Immunoglobulin Horseradish Peroxidase (HRP) (DAKO; Agilent Technologies). Signal amplification was necessary only for HSD3B detection and was carried out using the TSA Plus DNP (HRP) System (PerkinElmer, Waltham, MA, USA). Visualization was by 3,3’-diaminobenzidine (DAB) (DAKO Real™, Agilent Technologies) and sections were counterstained with hematoxylin. For double immunofluorescence of CYP11A1 and CYP21A2, sections were dewaxed, antigen-retrieved, blocked and probed with CYP11A1 primary antibody as above. Antibody detection was carried out with Goat anti-rabbit HRP (DAKO (P0450); 1:1000 in phosphate-buffered saline (PBS)) followed by use of the Tyramide-TSA™ Plus Fluorescein kit (PerkinElmer). Antigen retrieval was repeated in order to denature the first set of antibodies. Sections were then re-probed with CYP21A2 primary antibody as above with detection by Alexa Fluor 594 (red) goat anti-rabbit (1:1000 in TBS) and counterstained with ToPro®-3-iodide (Invitrogen; Select FX™ Nuclear Labeling Kit; S33025). Confocal images were captured using a Zeiss LSM 710 microscope and ZEN software (Carl Zeiss Microimaging, LLC, Thornwood, NY, USA).

### Western blotting

Proteins were separated by sodium dodecyl sulphate (SDS) gel electrophoresis using a XCell4 SureLock™ Midi-Cell apparatus (Life Technologies). Protein samples (30 μg) in NuPAGE® LDS Sample Buffer, containing NuPAGE® Sample Reducing Agent, were loaded to a NuPAGE® 4–12% Bis-Tris Midi Protein Gel (Life Technologies). Electrophoresis was carried out in NuPAGE® MOPS SDS Running Buffer, and the separated proteins were transferred to nitrocellulose using the Invitrogen iBlot® system and iBlot® Transfer Stacks (Life Technologies). Blocking was carried out using Odessey® Blocking Buffer (PBS; LI-COR, Cambridge, UK). Membranes were probed overnight with one of a number of primary antibodies diluted in Odyssey buffer. The primary antibodies used were anti-CYP11A1, anti-HSD3B, anti-CYP17A1 and anti-CYP21A2, as used for immunohistochemistry, and anti-β-actin (monoclonal mouse antibody (ab8226; Abcam, Cambridge, UK)). Bound antibody was detected with fluorescent secondary antibody (Cross Adsorbed, DyLight conjugated secondary antibodies; Thermo Fisher) and scanned using an Odyssey CLx-0565 Imager (LI-COR). Bands were quantified using Image Studio software (LI-COR).

### Statistical analysis

A general linear model was used to examine the effects of age, sex, smoking and any interactions between these factors on mRNA transcript, protein and steroid levels. The data were modelled by a quasi-Poisson distribution to account for over-dispersion. The minimal adequate model was used followed by Tukey’s post hoc analysis for factorial variables. Levene’s test was used to compare variability between groups. Statistical analysis was performed using R Studio (Boston, MA, USA). For statistical analysis of adrenal steroids, measurements below the LOQ for each steroid were substituted with a value of 0.

## Results

### Fetal plasma levels of adrenal-related hormones

**Fig. 1:**
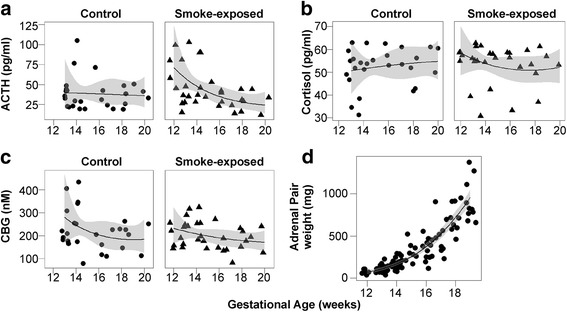
Fetal plasma ACTH, cortisol and CBG and adrenal weight during the second trimester. Data points from individual fetuses are shown (*circles* for control and *triangles* for smoke-exposed, where groups are separated). *Black lines* indicate generalized linear regression, and *grey fill* denotes the confidence interval (0.95) around an individual regression. There was no effect of fetal sex on levels of ACTH, cortisol or CBG (*n* = 60). The interaction between fetal smoke exposure and gestational age on ACTH levels (**a**) approached significance, however (*P* = 0.052). Levels of cortisol (**b**) and CBG (**c**) were unaffected by age or maternal smoking. Levels of CBG decreased between 12 and 20 weeks (**c**; *P* = 0.038). Combined fetal adrenal weights increased between 12 and 20 weeks of gestation (**d**; *P* < 0.001; *n* = 109) with no effect of fetal sex or maternal smoking

ACTH, cortisol and CBG were detectable in fetal plasma (using a sub-population of samples, see Table 1) throughout the second trimester (Fig. 1). Levels of ACTH and cortisol did not change significantly during this period, but CBG showed a significant decrease between 12 and 20 weeks of gestation (Fig. 1c; *P* = 0.038). Hormone levels did not differ significantly according to fetal sex. Maternal smoking was associated with altered levels of ACTH (Fig. 1a; interaction between age and smoking, *P* = 0.052) with levels generally higher towards the start of the second trimester. Levels of cortisol (Fig. 1b) and CBG (Fig. 1c) were similar between control and smoke-exposed fetuses.

### Fetal adrenal steroid content

**Fig. 2:**
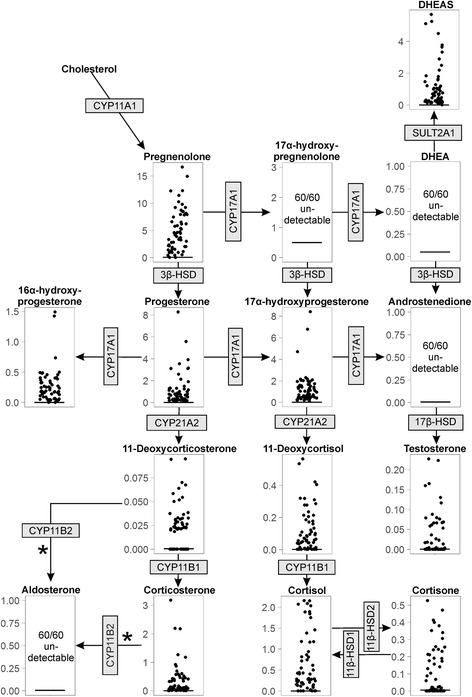
Intra-adrenal steroid levels in the human fetal adrenal during the second trimester. Steroid levels, expressed as ng/mg of tissue, are shown for each steroid measured (*n* = 60) and are arranged within the canonical steroidogenic pathways. The enzymes responsible for each conversion are shown in *grey boxes*. Data points from individual fetuses are shown, and the limit of quantitation for each steroid is shown as a *horizontal line*. Aldosterone, 17α-hydroxypregnenolone, DHEA and androstenedione were not detectable in any of the 60 samples. *The conversion of 11-deoxycorticosterone to aldosterone by CYP11B2 occurs via corticosterone and 18-hydroxycorticosterone as intermediates

**Fig. 3:**
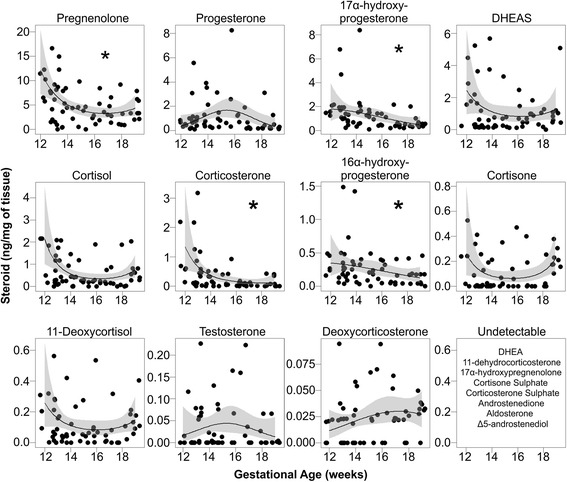
Changes in intra-adrenal steroid levels (ng/mg of tissue) during the second trimester. Data points from individual fetuses are shown (*n* = 60), and steroids which show a significant change with gestational age are indicated with an *asterisk*. Levels of pregnenolone (*P* = 0.004), 17α-hydroxyprogesterone (*P* < 0.001), 16α-hydroxyprogesterone (*P* = 0.02) and corticosterone (*P* < 0.001) decreased from 12 to 19 weeks. Generalized linear regressions are shown as *black lines* with the corresponding confidence intervals (0.95) in *grey*

Overall fetal adrenal weight increased exponentially during the second trimester (*P* < 0.001) with no difference between sexes or due to maternal smoking (Fig. 1d). Intra-adrenal steroid concentrations (measured in a sub-population of the total, see Table 1) during the same period are shown in Fig. 2, arranged within relevant steroidogenic pathways. Pregnenolone was the most abundant steroid found in the human fetal adrenal (Fig. 2) with high levels of 17α-hydroxyprogesterone, DHEAS and progesterone also present. Cortisol levels were variable but were present in all samples. In contrast, aldosterone was not detectable in any samples, although deoxycorticosterone and corticosterone were present. Androstenedione was also undetectable in all samples, although this may be related to a relatively high LOQ (Table 2). Testosterone was detected at low levels in 32 out of 60 samples (detailed in Table 2). Most adrenal steroids showed no significant change in concentration over the course of the second trimester (Fig. 3). The exceptions were pregnenolone (*P* = 0.004), 17α-hydroxyprogesterone (*P* < 0.001), 16α-hydroxyprogesterone (*P* = 0.02) and corticosterone (*P* < 0.001), which decreased significantly over the same period (Fig. 3). Since adrenal weight increases markedly during the second trimester, the total adrenal content of measured, detectable steroids increased over this period with the exception of corticosterone which was unchanged (Additional file 1: Figure S1). No differences between control male and female fetuses were seen.

**Fig. 4:**
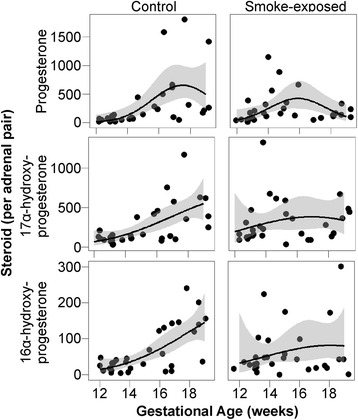
The effects of maternal smoking on intra-adrenal steroid levels during the second trimester. Measurements from individual fetuses are shown. Steroids are expressed as ng per total combined adrenal weight (*n* = 60). Maternal smoking was associated with altered intra-adrenal levels of three of the 19 steroids measured during the second trimester: progesterone (*P* = 0.02), 17α-hydroxyprogesterone (*P* = 0.02) and 16α-hydroxyprogesterone (*P* = 0.04) with *P* values corresponding to the interaction between smoke exposure and gestational age. *Black lines* show generalized linear regressions, and *grey fill* shows the confidence interval (0.95) around the regression

Maternal smoking was associated with altered intra-adrenal levels of progesterone (*P* = 0.02), 17α-hydroxyprogesterone (*P* = 0.02) and 16α-hydroxyprogesterone (*P* = 0.04) (Fig. 4; expressed per adrenal pair). Each of these steroids showed a significant interaction between smoking and gestational age across the second trimester.

### Tissue transcript and protein expression

#### mRNA transcripts

**Fig. 5:**
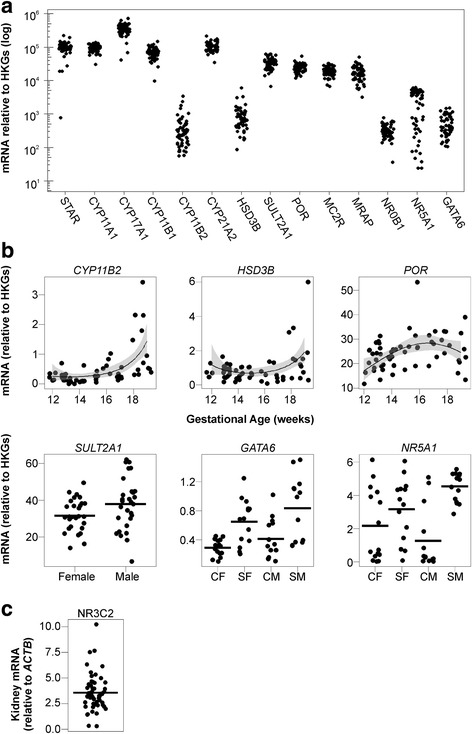
Expression levels of key mRNA transcripts in the human fetal adrenal and fetal kidney during the second trimester. **a** Overall adrenal transcript levels (relative to housekeeping genes (HKGs)) during the second trimester are shown on a log scale. Data points represent mRNA levels in individual fetuses. **b** Changes in adrenal transcript levels during the second trimester, due to fetal sex and/or maternal smoking. Expression of CYP11B2 (*P* = 0.003, *n* = 56) and HSD3B (*P* = 0.004, *n* = 58) increased between 12 and 19 weeks of gestation, while POR (*P* = 0.03, *n* = 60) increased during the initial phase of the trimester. Other transcripts did not change significantly during the second trimester. *Black lines* show the generalized linear regression, and *grey fill* denotes the confidence interval (0.95) around the regression. Levels of SULT2A1 were significantly different between male and female fetuses, *P* = 0.04 (*n* = 58), but there was no effect of maternal smoking (not shown). Maternal smoking was associated with increased levels of the transcription factor GATA6 (*P* < 0.001; *n* = 56) in both male and female fetuses and increased levels of NR5A1 (*P* = 0.04; *n* = 58) in male fetuses compared to control males. There were no other effects of age or smoking on transcript levels. *CF* control females, *SF* smoke-exposed females, *CM* control males, *SM* smoke-exposed males. **c** Transcript levels of the mineralocorticoid receptor NR3C2 in the human fetal kidney (*n* = 56; from 7 to 20 weeks of gestation). NR3C2 transcript expression did not differ significantly due to fetal age or sex, or maternal smoking. In all datasets, the *horizontal line* corresponds to the mean expression, and each data point corresponds to an individual fetus

Most enzymes in the adrenal steroidogenic pathway showed high levels of transcript expression throughout the second trimester (Fig. 5a). Levels of *CYP17A1, STAR*, *CYP21A2* and *CYP11A1*, all of which are all involved in the initial steps of steroid synthesis, were particularly high. In contrast, levels of *HSD3B* and *CYP11B2* transcripts were low but detectable. Transcripts encoding the ACTH receptor (MC2R), its accessory protein MRAP and the key steroidogenic transcription factors NR5A1 and GATA6 were also detectable in all adrenals investigated. Most transcripts showed no significant change in expression during the second trimester (Additional file 2: Figure S2) with the exceptions of *CYP11B2* (*P* = 0.003) and *HSD3B* (*P* = 0.004), which both increased during this period (Fig. 5b), and *POR* (*P* = 0.03), which increased during the initial part of the second trimester (Fig. 5b). Similarly, there were no sex differences in any of the transcript species measured apart from the key sulphation enzyme *SULT2A1*, which was significantly higher in males than in females (*P* = 0.04).

Maternal smoking was associated with increased expression of *GATA6* and *NR5A1* (Fig. 5b) in both sexes (*P* < 0.001). Furthermore, maternal smoking was also associated with an increase in the variability (Additional file 3: Figure S3) of *STAR* (*P* = 0.004) and *CYP17A1* (*P* = 0.02) in males.

Aldosterone acts primarily through the NR3C2 receptor in the kidney, and to determine whether the receptor is expressed during the second trimester, *NR3C2* levels were measured in human fetal kidneys. Transcript levels were variable but were detectable in all 56 human fetal samples tested (Fig. 5c). There were no significant differences in transcript levels due to gestational age, fetal sex or maternal smoking status.

#### Proteins

**Fig. 6:**
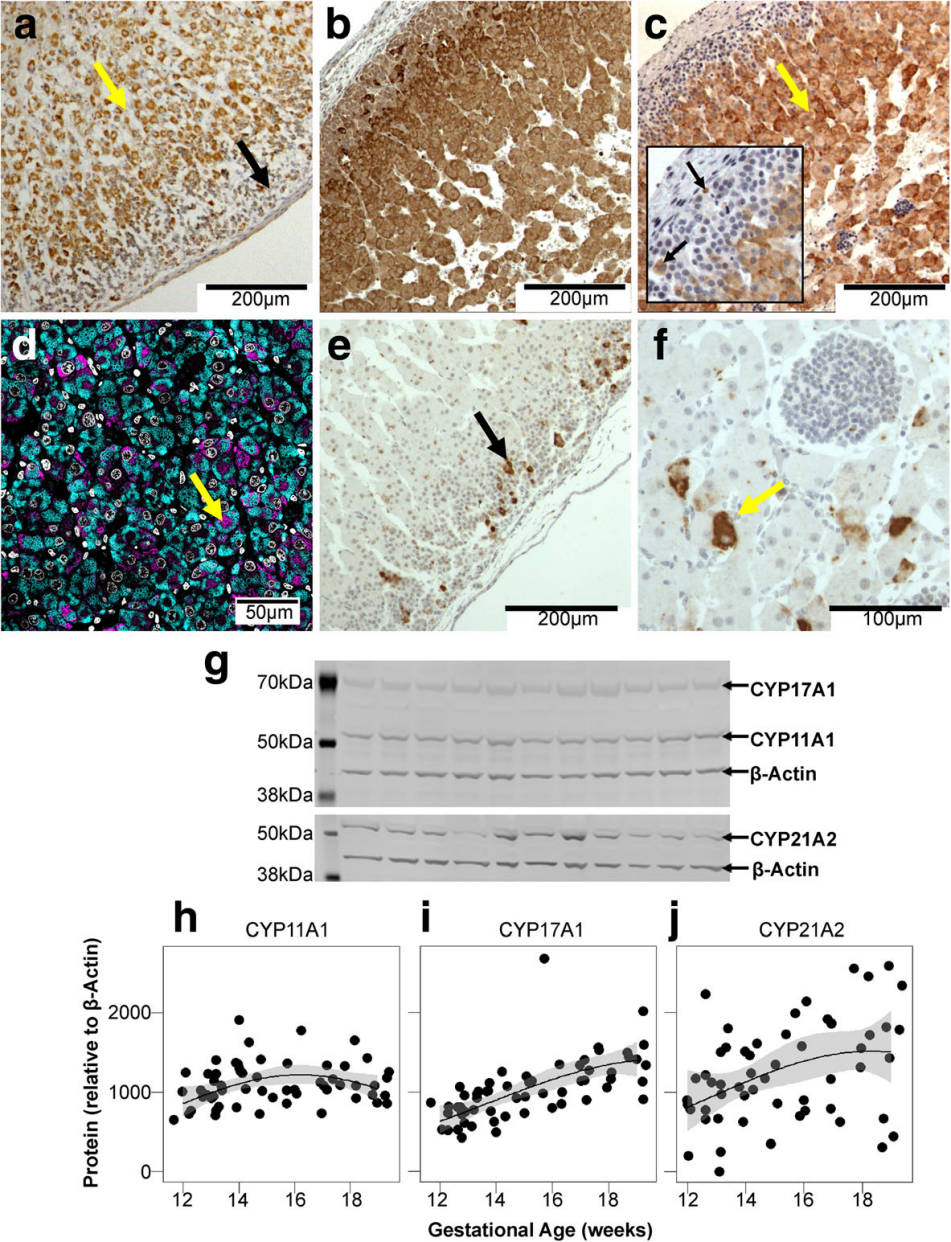
Steroidogenic enzyme localization and expression in the fetal adrenal. **a** CYP11A1 (*brown*) was localized primarily in the inner fetal zone (*yellow arrow*) with some cells in the definitive zone (*black arrow*) exhibiting weak immunostaining. **b** CYP17A1 (*brown*) was expressed throughout the adrenal, while (**c**) CYP21A2 (*brown*) was localized primarily in the fetal zone (*yellow arrow*), although protein expression was also seen in the definitive zone (*black arrows, inset*). **d** CYP11A1 (*cyan*) and CYP21A2 (*magenta*) co-localize in some cells of the fetal zone (*yellow arrow*), but most cells only express one of these enzymes with CYP21A2 the predominant enzyme. **e** HSD3B was predominantly localized to the outer definitive zone (*black arrow*), although it was also present in some cells within the fetal zone of adrenals at 12 weeks of gestation (**f**; *yellow arrow*). These HSD3B-positive cells in the fetal zone were not observed after 13 weeks of gestation. **g** Representative Western blot showing CYP11A1, CYP17A1, CYP21A2 and the loading control β-actin. Molecular weight markers are labelled on the *left* of the image. **h**–**j** Expression levels of CYP11A1, CYP17A1 and CYP21A2 protein in the fetal adrenal during the second trimester. Data points from individual fetuses are shown. *Black lines* show the generalized linear regression, and *grey fill* denotes the confidence interval (0.95) around the regression. Expression of CYP11A1 was unaffected by gestational age, while both CYP17A1 (*P* = 0.004) and CYP21A2 (*P* = 0.04) showed increased expression between 12 and 19 weeks

CYP11A1 (Fig. 6a and d) was primarily localized in the adrenal fetal zone and transitional zone, with some immunoreactive cells also present in the definitive zone. CYP17A1 was localized to the fetal zone and transition zone (Fig. 6b), while CYP21A2 was most prominent in the fetal zone although also detectable in the definitive zone (Fig. 6c and d). CYP11A1 and CYP21A2 were co-localized in some cells in the fetal zone, although many cells expressed CYP21A2 alone while a few expressed only CYP11A1 (Fig. 6d).

HSD3B was present primarily in cells of the adrenal definitive zone (Fig. 6e), although a small number of cells in the fetal zone in younger fetuses (12–13 weeks; Fig. 6f) also expressed the protein. These cells were comparable in size to the other cells of the fetal zone and were larger than the densely packed cells of the definitive zone. HSD3B-expressing cells in the fetal zone were not observed in the adrenals of fetuses older than 13 weeks.

Adrenal levels of CYP11A1, CYP17A1 and CYP21A2 protein, measured using Western blotting (Fig. 6g), were similar between males and females, and no significant differences were observed between control and smoke-exposed groups. Levels of CYP11A1 were not significantly altered by gestational age (Fig. 6h), whereas concentrations of both CYP17A1 (Fig. 6i) and CYP21A2 (Fig. 6j) increased significantly between 12 and 19 weeks of gestation, regardless of sex or smoke exposure (*P* = 0.04, and *P* = 0.004 respectively). HSD3B was barely detectable by Western blotting and could not be reliably quantified (not shown).

## Discussion

The human fetal adrenal cortex and the placenta regulate most aspects of fetal steroid endocrinology, together with the fetal liver and testes [25]. Normal development of the adrenal gland is therefore essential in maintaining fetal levels of glucocorticoids, mineralocorticoids and estrogens. Failure of normal adrenal development can lead to congenital adrenal hyperplasia (most commonly through loss of 21-hydroxylase activity and leading to disorders of sex development [26, 27]), salt-wasting disorders and adrenal insufficiency in the newborn [3, 12, 28, 29]. In this study, we have used a large cohort (up to 109 fetuses) of well-characterized, normal, human fetal samples to detail changes in fetal adrenal steroidogenesis during the second trimester, a critical time for fetal growth, development and sex differentiation. We show that the fetal adrenal produces cortisol throughout the second trimester, possibly from placental progesterone [27]. However, we also show that aldosterone is not synthesized in detectable amounts during this period, which may be of importance in understanding pre-term neonatal salt wasting. Maternal smoking changes fetal ACTH levels, but this is not accompanied by down-stream effects on the primary adrenal steroids.

Together, the concentrations of intra-adrenal steroids and expression of steroidogenic enzymes indicate that the fetal adrenal is highly active throughout the second trimester in the human. In our studies, the enzymes necessary for adrenal steroid synthesis were expressed by week 12 of gestation and mostly at relatively high levels apart from *HSD3B* and *CYP11B2*, which were low at this stage. Low levels of *HSD3B* and *CYP11B2* transcripts in the late second/early third trimester have also been reported previously [9, 30]. Despite low HSD3B transcript and protein expression, however, the Δ^4^ steroids progesterone, 17α-hydroxyprogesterone and cortisol were present in significant amounts throughout the second trimester. The fetal adrenals also generated significant amounts of 16α-hydroxyprogesterone, as reported previously [31, 32], due to the relatively high 16α-hydroxylation activity of human CYP17A1 [33]. The presence of these Δ^4^ steroids in such high amounts may occur because HSD3B enzyme activity does not reflect transcript or protein levels or because placental progesterone, derived from the circulation, is being converted in the fetal adrenals as previously suggested [13, 34]. Elevated amniotic fluid levels of 17α-hydroxyprogesterone have been reported to be a reasonable indicator of 21-hydroxylase deficiency and congenital adrenal hyperplasia in the mid-gestation fetus [35, 36]. While molecular methods are now more commonly used to diagnose congenital adrenal hyperplasia [37], our results show that fetal adrenal steroid concentrations do not change markedly after 12 weeks. It is, therefore, possible that amniotic fluid from early second trimester fetuses could be used to confirm diagnosis of 21-hydroxylase deficiency.

Salt-wasting disorders frequently occur in extreme pre-term neonates [38], and it has been suggested that this may be due to low expression of the mineralocorticoid receptor (NR3C2) in the pre-term kidney [39]. The results described here suggest, however, that neonatal salt-wasting syndrome in pre-term infants may occur because of a limited capacity of the fetal adrenal to generate aldosterone. This is consistent with earlier studies showing that tissue from second trimester human adrenals has little or no capability to produce aldosterone in vitro from tritiated substrate [40, 41]. It is also consistent with reports that pre-term neonates (26–32 weeks gestation) have normal sensitivity to aldosterone but reduced capacity to secrete the steroid [5] and with the demonstration (Fig. 5c) that the human fetal kidney consistently expresses *NR3C2* during the late first and the second trimester. Both CYP11B2 (aldosterone synthase) and CYP11B1 can catalyse the conversion of 11-deoxycorticosterone to corticosterone, although further conversion to aldosterone, via the intermediate 18-hydroxycorticosterone, is only performed by CYP11B2 [42–44]. Therefore, the absence of detectable fetal adrenal aldosterone in this study is likely a reflection of the low expression of CYP11B2 as previously predicted [13].

Human fetal adrenal glucocorticoid and androgen production is dependent on ACTH released by the fetal pituitary during the second trimester [13]. It has also been proposed that expression of the enzyme HSD3B and interaction with fetal ACTH (through altered negative feedback) is key to understanding the regulation of fetal adrenal steroid production [1]. Goto et al. [1] have reported that transiently elevated HSD3B expression occurs towards the end of the first trimester, leading to early cortisol biosynthesis. They suggest that this has a negative feedback effect on the fetal pituitary, reducing ACTH stimulation of fetal adrenal androgen secretion and thereby protecting female fetuses from masculinization. Our results extend the work of Goto et al [1] into the second trimester and show that the fetal adrenal continues to make cortisol throughout this period, despite the loss of HSD3B activity. This means that ACTH will remain under negative feedback control during the second trimester. One complication of the hypothesis of Goto et al. [1] is that higher HSD3B activity during the first trimester will tend to favour androgen production, which will then be in a balance between enzyme activity and altered negative feedback. This is particularly pertinent as human CYP17A1 has poor C17-20 lyase activity with 17α-hydroxyprogesterone as substrate [44], meaning that adrenal Δ^4^ androgens are only likely to be generated de novo (under ACTH regulation) rather than from placental progesterone. Adrenal androstenedione is present at the end of the first trimester at relatively high levels [1, 11] but not in the second trimester. This suggests that adrenal Δ^4^ androgen production, by both sexes, may be higher during the first trimester, reflecting HSD3B activity rather than altered negative feedback.

The presence of adrenal cortisol throughout the second trimester also has implications for treatment of congenital adrenal hyperplasia. This condition arises through deficiency of one of the enzymes involved in cortisol synthesis, commonly 21-hydroxylase. In turn, this leads to increased ACTH levels through impaired negative feedback and to increased adrenal androgen production. The condition is normally treated through administration of dexamethasone from the first trimester until birth. It has been suggested, however, that dexamethasone treatment in these cases may only be necessary in the first half of pregnancy [45]. One proposed reason is that fetal cortisol is absent (based on HSD3B expression) during the second trimester and that fetal ACTH would not normally be under negative feedback control during this period. The demonstration here that the fetal adrenal produces cortisol throughout the second trimester is of direct relevance, therefore, and suggests that normal homeostatic feedback regulation remains in place at all times after the first trimester.

One possible confounding factor in these studies is the effect of the termination regime on fetal ACTH and steroid levels. RU486 acts as an antagonist at both the progesterone and glucocorticoid receptors, and in non-pregnant subjects daily administration will increase ACTH and cortisol over a 7-day period [46]. The dose of RU486 used clinically in this study, however, was 200 mg/patient (average 2.7 mg/kg), which is less than the dose required for anti-glucocorticoid action (4.5 mg/kg) [47]. Furthermore, while RU486 transfers readily across the placenta, fetal plasma concentrations of the drug are 0.1–10% of those found in the maternal circulation [48, 49], and it is highly unlikely that fetal drug concentrations will reach the threshold levels required to affect the fetal HPA axis. This is borne out by an earlier study which reported that use of RU486 during terminations (at 600 mg, three times the dose used in this study) had no effect on fetal or maternal plasma cortisol concentrations [48]. Similarly, RU486 is reported to have no effect on placental ACTH during first or second trimester termination [50].

The adrenal gland is highly zonated both morphologically and functionally. Earlier studies by Jaffe and colleagues [13, 51–53] developed the concept, based on spatial distribution of steroidogenic enzymes, that the human fetal adrenal in the third trimester is composed of three functional zones which are analogous to the adult cortical zones. They proposed that the definitive, transitional and fetal zones are analogous to the zona glomerulosa, the zona fasciculata and the zona reticularis respectively. Our immunolocalization results generally agree with these and other studies [7, 9, 10, 13, 51–53] and suggest that functional zonation is present from as early as 12 weeks. These immunolocalization studies show that the fetal zone is the main site of CYP11A1, CYP17A1 and CYP21A2 expression during the second trimester. This means that the fetal zone is likely to be the main site of DHEA production and of de novo steroid synthesis. Synthesis of other adrenal steroids requires HSD3B activity, however, which is localized primarily in the definitive zone. This means either that steroid intermediates move between the adrenal zones to facilitate de novo synthesis or that Δ^4^ steroid synthesis depends largely on placental progesterone as described above. Given the low levels of adrenal HSD3B during the second trimester and the intense vascularization of the fetal zone, which would favour delivery of steroid substrate from the circulation, use of placental progesterone appears the more likely route [34]. The presence of all components of the steroidogenic pathway in the adrenal also means, however, that de novo synthesis is likely to contribute to overall steroid levels and to androgen levels in particular, as discussed above. Our co-localization studies show that, within the fetal zone, different cells express different combinations of steroidogenic enzymes. This may be because they represent different populations of cells, or it possibly may be due to the local environment of the cell.

The effect of maternal smoking on fetal adrenal function and HPA axis development is most clearly seen in the association between maternal smoking and sudden infant death syndrome [54], which may be linked to adrenal dysfunction [55]. Our results show that maternal smoking is associated with altered circulating fetal ACTH (*P* = 0.052), although there was no effect on plasma cortisol levels. Intra-adrenal fetal cortisol concentrations were also unaffected by smoke exposure, which suggests that there is a reduced sensitivity of the adrenal to ACTH through down-regulation of either the receptor or second messenger systems. These data are consistent with a previous study showing increased ACTH, but unchanged cortisol, at birth following exposure to maternal smoking in utero [16]. These programmed changes in adrenal sensitivity to ACTH induced by maternal smoking may be of importance after birth if ACTH levels return to normal in exposed neonates but adrenal sensitivity remains reduced. Consistent with this hypothesis, neonatal cortisol levels and cortisol response to stress are reported to be significantly reduced in the first month after birth following exposure to maternal smoking in utero [56].

Maternal smoking is also associated with altered whole-adrenal levels (nanograms/adrenal pair) of progesterone, 17α-hydroxyprogesterone and 16α-hydroxyprogesterone across the second trimester. As discussed above, it is likely that Δ^4^ steroids in the fetal adrenal are derived, at least in part, from placental progesterone, and so these changes may reflect alterations in placental activity in response to maternal smoking. A number of studies have shown that maternal smoking can alter placental function and may reduce placental progesterone concentrations [57]. We have reported that circulating maternal progesterone is not clearly affected by maternal smoking [58]. However, if plasma concentrations in the fetus more closely represent placental production, then this may have a knock-on effect on total adrenal steroid levels.

The most marked effects of maternal smoking on the fetal adrenal were increased levels of *NR5A1* and *GATA6* transcripts. *NR5A1* encodes steroidogenic factor 1 (SF-1), which is a nuclear receptor transcription factor essential for development and function of the adrenals and gonads [8]. Disruption of SF-1, unless severe, has greater clinical effects on the gonads than the adrenals [59], but it is unclear what effects overexpression of SF-1 in utero will have on adrenal development in the human. Overexpression of SF-1 in mice leads to an increase in adrenal size through increased commitment of precursors to the steroidogenic lineage [14, 60], although maternal smoking had no significant effects on human fetal adrenal weight in our study. It remains to be seen, however, whether the morphological and cellular composition of the fetal adrenals is affected by smoking. GATA6 is a zinc finger transcription factor which works in concert with SF-1 [61] to activate steroidogenic enzyme expression in general and HSD3B in particular [62–64]. While no significant changes in steroidogenic enzyme expression were seen in the smoke-exposed group, the increased variability in *STAR* and *CYP17A1* expression may be related to altered transcription factor expression.

## Conclusions

Along with the placenta, the fetal adrenal acts to regulate fetal steroid endocrinology, and normal fetal development of the adrenals is essential for post-natal health. Mapping the interactions between placenta, adrenals and other endocrine organs (e.g. the testes) is essential, therefore, to understand how these processes can be dysregulated. This study is the first to comprehensively document the changes in adrenal steroid levels and steroidogenic enzyme expression throughout the second trimester and shows that a combination of substrate availability, enzyme expression and enzyme specificity regulates adrenal steroid production. Critically, this study shows that the fetal adrenals do not synthesize detectable levels of aldosterone, which is likely to explain pre-term salt-wasting conditions. They do, however, synthesize cortisol throughout the second trimester, which indicates that fetal ACTH secretion is under inhibitory regulation by the adrenal during this period with implications for our understanding and treatment of congenital adrenal hyperplasia. Maternal smoking did not have marked effects on fetal adrenal steroidogenic function, but it did alter fetal ACTH levels, which may trigger homeostatic mechanisms in the adrenal leading to knock-on effects in the neonate.

## Additional files

Additional file 1: Figure S1. Changes in whole intra-adrenal steroid levels (ng/adrenal pair) during the second trimester. Data points from individual fetuses are shown (*n* = 60). All detectable steroid levels increase significantly with gestational age (*P* < 0.001) with the exception of corticosterone. Generalized linear regressions are shown as *black lines* with the corresponding confidence intervals (0.95) in *grey*. (TIF 80 kb)

Additional file 2: Figure S2. Age-dependent changes in adrenal expression of mRNA transcripts encoding factors involved in steroid synthesis. *CYP11B2*, *HSD3B* and *POR* are also shown in Fig. 5 and have been included here for completeness. Transcript levels are plotted relative to housekeeping genes (HKGs), and each point represents data from an individual fetus. *Black lines* denote a generalized linear regression, and *grey fill* denotes the confidence interval (0.95) around the regression. (TIF 310 kb)

Additional file 3: Figure S3. Effect of maternal smoking on *STAR* and *CYP17A1* transcript levels in the fetal adrenal during the second trimester. Maternal smoking was associated with an increase in the variability (Levene’s test) of transcript expression of *STAR* (*P* = 0.004) and *CYP17A1* (*P* = 0.02), in male smoke-exposed (SE) fetuses compared to male controls (C). Each point represents data from an individual fetus and transcript levels are expressed relative to HKGs. *Black lines* denote a generalized linear regression, and *grey fill* denotes the confidence interval (0.95) around the regression. Data points for females are shown in the *left* of each panel and males on the *right*. Data points for controls are shown on the top of each panel and smoke-exposed on the bottom. (TIF 339 kb)

## Abbreviations

ACTH: Adrenocorticotropic hormone
CBG: Corticosteroid-binding globulin
CRL: Crown-rump length
DHEA: Dehydroepiandrosterone
DHEAS: Dehydroepiandrosterone sulphate
HPA: Hypothalamo-pituitary-adrenal
IS: Internal standard
LOD: Limit of detection
LOQ: Limit of quantitation
PMM1: Phosphomannomutase 1
RSD: Relative standard deviation
S/N: Signal-to-noise ratio
SEM: Standard error of the mean
SF-1: Steroidogenic factor 1
TBP: TATA box binding protein

## References

[1] Goto M, Hanley KP, Marcos J, Wood PJ, Wright S, Postle AD, Cameron IT, Mason JI, Wilson DI, Hanley NA (2006). In humans, early cortisol biosynthesis provides a mechanism to safeguard female sexual development. J Clin Investig.

[2] Rainey WE, Rehman KS, Carr BR (2004). The human fetal adrenal: making adrenal androgens for placental estrogens. Semin Reprod Med.

[3] Lekarev O, New MI (2011). Adrenal disease in pregnancy. Best Pract Res Clin Endocrinol Metab.

[4] Seckl JR (2001). Glucocorticoid programming of the fetus; adult phenotypes and molecular mechanisms. Mol Cell Endocrinol.

[5] Martinerie L, Pussard E, Yousef N, Cosson C, Lema I, Husseini K, Mur S, Lombes M, Boileau P (2015). Aldosterone-signaling defect exacerbates sodium wasting in very preterm neonates: the Premaldo Study. J Clin Endocrinol Metab.

[6] Fernandez EF, Watterberg KL (2009). Relative adrenal insufficiency in the preterm and term infant. J Perinatol.

[7] Narasaka T, Suzuki T, Moriya T, Sasano H (2001). Temporal and spatial distribution of corticosteroidogenic enzymes immunoreactivity in developing human adrenal. Mol Cell Endocrinol.

[8] Hanley NA, Rainey WE, Wilson DI, Ball SG, Parker KL (2001). Expression profiles of SF-1, DAX1, and CYP17 in the human fetal adrenal gland: potential interactions in gene regulation. Mol Endocrinol.

[9] Naccache A, Louiset E, Duparc C, Laquerriere A, Patrier S, Renouf S, Gomez-Sanchez CE, Mukai K, Lefebvre H, Castanet M (2016). Temporal and spatial distribution of mast cells and steroidogenic enzymes in the human fetal adrenal. Mol Cell Endocrinol.

[10] Folligan K, Bouvier R, Targe F, Morel Y, Trouillas J (2005). Histological and molecular study of fetal human adrenal cortex (12-36 wk). Ann Endocrinol (Paris).

[11] Savchuk I, Morvan ML, Antignac JP, Gemzell-Danielsson K, Le Bizec B, Soder O, Svechnikov K (2017). Androgenic potential of human fetal adrenals at the end of the first trimester. Endocr Connect.

[12] Ishimoto H, Jaffe RB (2011). Development and function of the human fetal adrenal cortex: a key component in the feto-placental unit. Endocr Rev.

[13] Mesiano S, Jaffe RB (1997). Developmental and functional biology of the primate fetal adrenal cortex. Endocr Rev.

[14] Cornelius MD, Day NL (2009). Developmental consequences of prenatal tobacco exposure. Curr Opin Neurol.

[15] O'Shaughnessy PJ, Monteiro A, Bhattacharya S, Fowler PA (2011). Maternal smoking and fetal sex significantly affect metabolic enzyme expression in the human fetal liver. J Clin Endocrinol Metab.

[16] McDonald SD, Walker M, Perkins SL, Beyene J, Murphy K, Gibb W, Ohlsson A (2006). The effect of tobacco exposure on the fetal hypothalamic–pituitary–adrenal axis. Br J Obstet Gynaecol.

[17] Kanaka-Gantenbein C (2010). Fetal origins of adult diabetes. Ann N Y Acad Sci.

[18] Xita N, Tsatsoulis A (2010). Fetal origins of the metabolic syndrome. Ann N Y Acad Sci.

[19] O'Shaughnessy PJ, Baker PJ, Monteiro A, Cassie S, Bhattacharya S, Fowler PA (2007). Developmental changes in human fetal testicular cell numbers and messenger ribonucleic acid levels during the second trimester. J Clin Endocrinol Metab.

[20] Hammond GL, Lähteenmäki PLA (1983). A versatile method for the determination of serum cortisol binding globulin and sex hormone binding globulin binding capacities. Clin Chim Acta.

[21] Schloms L, Storbeck KH, Swart P, Gelderblom WC, Swart AC (2012). The influence of Aspalathus linearis (Rooibos) and dihydrochalcones on adrenal steroidogenesis: quantification of steroid intermediates and end products in H295R cells. J Steroid Biochem Mol Biol.

[22] O'Shaughnessy PJ, Willerton L, Baker PJ (2002). Changes in Leydig cell gene expression during development in the mouse. Biol Reprod.

[23] Baker PJ, O'Shaughnessy PJ (2001). Expression of prostaglandin D synthetase during development in the mouse testis. Reproduction.

[24] Andersen CL, Jensen JL, Orntoft TF (2004). Normalization of real-time quantitative reverse transcription-PCR data: a model-based variance estimation approach to identify genes suited for normalization, applied to bladder and colon cancer data sets. Cancer Res.

[25] O'Shaughnessy PJ, Monteiro A, Bhattacharya S, Fraser MJ, Fowler PA (2013). Steroidogenic enzyme expression in the human fetal liver and potential role in the endocrinology of pregnancy. Mol Hum Reprod.

[26] Kamrath C, Hochberg Z, Hartmann MF, Remer T, Wudy SA (2012). Increased activation of the alternative “backdoor” pathway in patients with 21-hydroxylase deficiency: evidence from urinary steroid hormone analysis. J Clin Endocrinol Metab.

[27] Fukami M, Homma K, Hasegawa T, Ogata T (2013). Backdoor pathway for dihydrotestosterone biosynthesis: implications for normal and abnormal human sex development. Dev Dyn.

[28] El-Maouche D, Arlt W, Merke DP (2017). Congenital adrenal hyperplasia. Lancet.

[29] Witchel SF, Azziz R (2011). Congenital adrenal hyperplasia. J Pediatr Adolesc Gynecol.

[30] Pezzi V, Mathis JM, Rainey WE, Carr BR (2003). Profiling transcript levels for steroidogenic enzymes in fetal tissues. J Steroid Biochem Mol Biol.

[31] Swart P, Swart AC, Waterman MR, Estabrook RW, Mason JI (1993). Progesterone 16 alpha-hydroxylase activity is catalyzed by human cytochrome P450 17 alpha-hydroxylase. J Clin Endocrinol Metab.

[32] Arlt W, Martens JW, Song M, Wang JT, Auchus RJ, Miller WL (2002). Molecular evolution of adrenarche: structural and functional analysis of p450c17 from four primate species. Endocrinology.

[33] Storbeck KH, Kolar NW, Stander M, Swart AC, Prevoo D, Swart P (2008). The development of an ultra performance liquid chromatography-coupled atmospheric pressure chemical ionization mass spectrometry assay for seven adrenal steroids. Anal Biochem.

[34] Macnaughton MC, Taylor T, McNally EM, Coutts JR (1977). The effect of synthetic ACTH on the metabolism of [4-14C]-progesterone by the previable human fetus. J Steroid Biochem.

[35] Wudy SA, Dorr HG, Solleder C, Djalali M, Homoki J (1999). Profiling steroid hormones in amniotic fluid of midpregnancy by routine stable isotope dilution/gas chromatography-mass spectrometry: reference values and concentrations in fetuses at risk for 21-hydroxylase deficiency. J Clin Endocrinol Metab.

[36] Forest MG, Betuel H, Couillin P, Boue A (1981). Prenatal diagnosis of congenital adrenal hyperplasia (CAH) due to 21-hydroxylase deficiency by steroid analysis in the amniotic fluid of mid-pregnancy: comparison with HLA typing in 17 pregnancies at risk for CAH. Prenat Diagn.

[37] Tardy-Guidollet V, Menassa R, Costa JM, David M, Bouvattier-Morel C, Baumann C, Houang M, Lorenzini F, Philip N, Odent S (2014). New management strategy of pregnancies at risk of congenital adrenal hyperplasia using fetal sex determination in maternal serum: French cohort of 258 cases (2002-2011). J Clin Endocrinol Metab.

[38] Barrington KJ (2014). Management during the first 72 h of age of the periviable infant: an evidence-based review. Semin Perinatol.

[39] Martinerie L, Viengchareun S, Delezoide AL, Jaubert F, Sinico M, Prevot S, Boileau P, Meduri G, Lombes M (2009). Low renal mineralocorticoid receptor expression at birth contributes to partial aldosterone resistance in neonates. Endocrinology.

[40] Nelson HP, Kuhn RW, Deyman ME, Jaffe RB (1990). Human fetal adrenal definitive and fetal zone metabolism of pregnenolone and corticosterone: alternate biosynthetic pathways and absence of detectable aldosterone synthesis. J Clin Endocrinol Metabol.

[41] Dufau ML, Villee DB (1969). Aldosterone biosynthesis by human fetal adrenal in vitro. Biochem Biophys Acta.

[42] Portrat-Doyen S, Tourniaire J, Richard O, Mulatero P, Aupetit-Faisant B, Curnow KM, Pascoe L, Morel Y (1998). Isolated aldosterone synthase deficiency caused by simultaneous E198D and V386A mutations in the CYP11B2 gene. J Clin Endocrinol Metabol.

[43] Schiffer L, Anderko S, Hannemann F, Eiden-Plach A, Bernhardt R (2015). The CYP11B subfamily. J Steroid Biochem Mol Biol.

[44] Strushkevich N, Gilep AA, Shen L, Arrowsmith CH, Edwards AM, Usanov SA, Park HW (2013). Structural insights into aldosterone synthase substrate specificity and targeted inhibition. Mol Endocrinol.

[45] Hanley NA, Arlt W (2006). The human fetal adrenal cortex and the window of sexual differentiation. Trends Endocrinol Metab.

[46] Block T, Petrides G, Kushner H, Kalin N, Belanoff J, Schatzberg A (2017). Mifepristone plasma level and glucocorticoid receptor antagonism associated with response in patients with psychotic depression. J Clin Psychopharmacol.

[47] Mifegyne. http://www.medicines.org.uk/emc/medicine/617. Accessed 7 Dec 2017.

[48] Hill NC, Selinger M, Ferguson J, MacKenzie IZ (1990). The placental transfer of mifepristone (RU 486) during the second trimester and its influence upon maternal and fetal steroid concentrations. Br J Obstet Gynaecol.

[49] Ishii A, Zaitsu K, Kusano M, Asano T, Ogawa T, Hattori H, Seno H (2015). Identification and quantitation of mifepristone and its N-demethyl metabolite in the plasma of an aborted fetus by liquid chromatography–quadrupole–time-of-flight–mass spectrometry (LC–Q–TOFMS) and ultra-performance liquid chromatography–tandem mass spectrometry (UPLC–MS–MS). Forensic Toxicol.

[50] Cooper ES, Greer IA, Brooks AN (1996). Placental proopiomelanocortin gene expression, adrenocorticotropin tissue concentrations, and immunostaining increase throughout gestation and are unaffected by prostaglandins, antiprogestins, or labor. J Clin Endocrinol Metab.

[51] Coulter CL, Jaffe RB (1998). Functional maturation of the primate fetal adrenal in vivo: 3. Specific zonal localization and developmental regulation of CYP21A2 (P450c21) and CYP11B1/CYP11B2 (P450c11/aldosterone synthase) lead to integrated concept of zonal and temporal steroid biosynthesis. Endocrinology.

[52] Mesiano S, Coulter CL, Jaffe RB (1993). Localization of cytochrome P450 cholesterol side-chain cleavage, cytochrome P450 17 alpha-hydroxylase/17, 20-lyase, and 3 beta-hydroxysteroid dehydrogenase isomerase steroidogenic enzymes in human and rhesus monkey fetal adrenal glands: reappraisal of functional zonation. J Clin Endocrinol Metab.

[53] Coulter CL, Goldsmith PC, Mesiano S, Voytek CC, Martin MC, Mason JI, Jaffe RB (1996). Functional maturation of the primate fetal adrenal in vivo. II. Ontogeny of corticosteroid synthesis is dependent upon specific zonal expression of 3 beta-hydroxysteroid dehydrogenase/isomerase. Endocrinology.

[54] Fleming P, Blair PS (2007). Sudden Infant Death Syndrome and parental smoking. Early Hum Dev.

[55] Gozzi TG, Harris NP, McGown IN, Cowley DM, Cotterill AM, Campbell PE, Anderson PK, Warne GL (2005). Autopsy diagnosis of 21-hydroxylase deficiency CAH in a case of apparent SIDS. Pediatr Dev Pathol.

[56] Stroud LR, Papandonatos GD, Rodriguez D, McCallum M, Salisbury AL, Phipps MG, Lester B, Huestis MA, Niaura R, Padbury JF, Marsit CJ (2014). Maternal smoking during pregnancy and infant stress response: test of a prenatal programming hypothesis. Psychoneuroendocrinology.

[57] Piasek M, Blanuša M, Kostial K, Laskey JW (2001). Placental cadmium and progesterone concentrations in cigarette smokers. Reprod Toxicol.

[58] Huuskonen P, Amezaga MR, Bellingham M, Jones LH, Storvik M, Hakkinen M, Keski-Nisula L, Heinonen S, O'Shaughnessy PJ, Fowler PA, Pasanen M (2016). The human placental proteome is affected by maternal smoking. Reprod Toxicol.

[59] Suntharalingham JP, Buonocore F, Duncan AJ, Achermann JC (2015). DAX-1 (NR0B1) and steroidogenic factor-1 (SF-1, NR5A1) in human disease. Best Pract Res Clin Endocrinol Metab.

[60] Zubair M, Oka S, Parker KL, Morohashi K (2009). Transgenic expression of Ad4BP/SF-1 in fetal adrenal progenitor cells leads to ectopic adrenal formation. Mol Endocrinol.

[61] Tremblay JJ, Viger RS (2003). Novel roles for GATA transcription factors in the regulation of steroidogenesis. J Steroid Biochem Mol Biol.

[62] Martin LJ, Tremblay JJ (2005). The human 3β-hydroxysteroid dehydrogenase/Δ^5^-Δ^4^ isomerase type 2 promoter is a novel target for the immediate early orphan nuclear receptor Nur77 in steroidogenic cells. Endocrinology.

[63] Mamluk R, Greber Y, Meidan R (1999). Hormonal regulation of messenger ribonucleic acid expression for steroidogenic factor-1, steroidogenic acute regulatory protein, and cytochrome P450 side-chain cleavage in bovine luteal cells. Biol Reprod.

[64] Jimenez P, Saner K, Mayhew B, Rainey WE (2003). GATA-6 is expressed in the human adrenal and regulates transcription of genes required for adrenal androgen biosynthesis. Endocrinology.

