# Supplementary figures and images for: The human fetal adrenal produces cortisol but no detectable aldosterone throughout the second trimester
Source: BMC Med. 2018 Feb 12;16:23. doi: 10.1186/s12916-018-1009-7 (PMC5808459; doi:10.1186/s12916-018-1009-7)

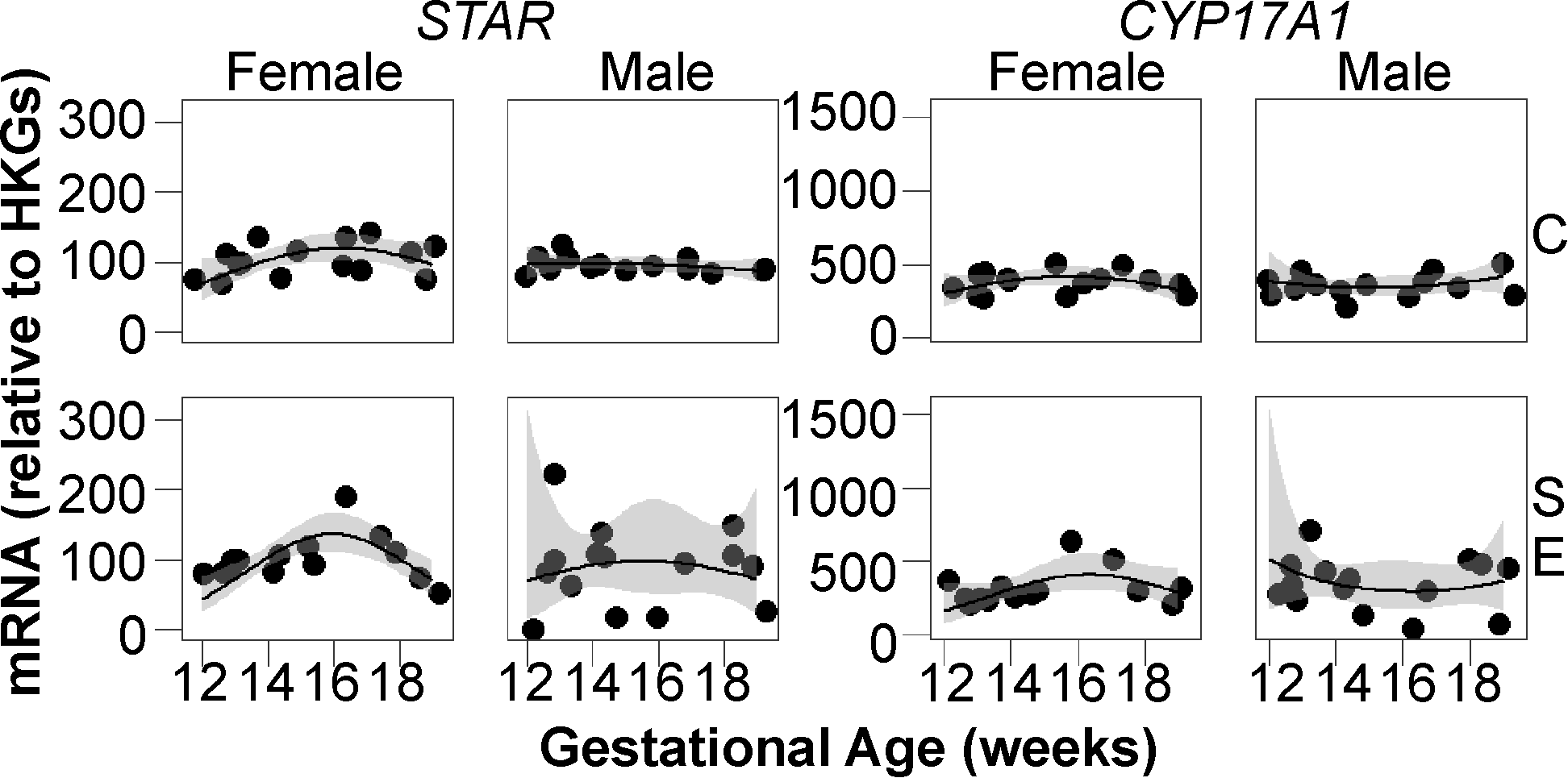

Supplement: Supplementary file 1 — Changes in whole intra-adrenal steroid levels (ng/adrenal pair) during the second trimester. Data points from individual fetuses are shown (n = 60). All detectable steroid levels increase significantly with gestational age (P < 0.001) with the exception of corticosterone. Generalized linear regressions are shown as black lines with the corresponding confidence intervals (0.95) in grey. (TIF 80 kb) [file 12916_2018_1009_MOESM1_ESM.tif]

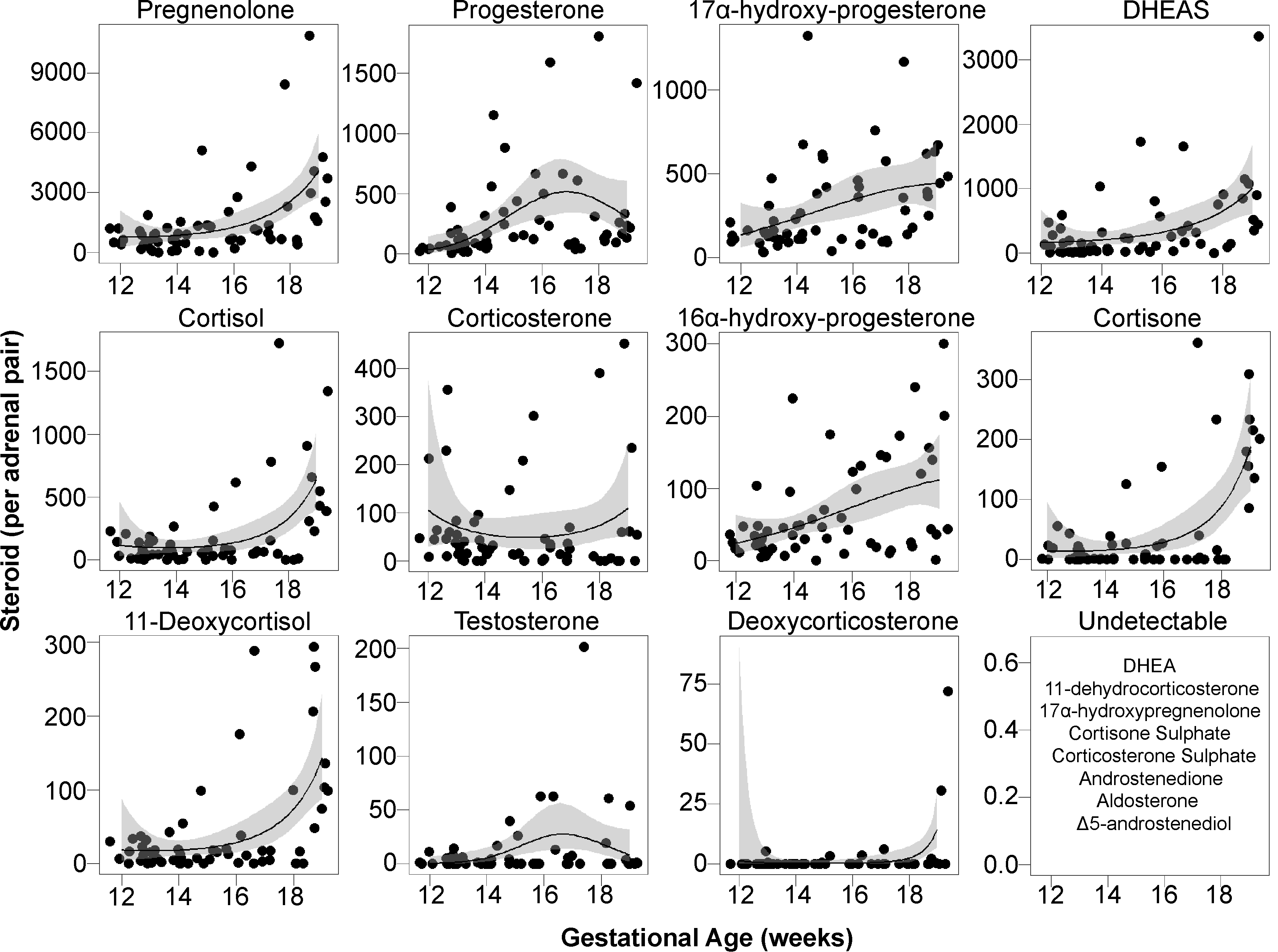

Supplement: Supplementary file 2 — Age-dependent changes in adrenal expression of mRNA transcripts encoding factors involved in steroid synthesis. CYP11B2, HSD3B and POR are also shown in Fig. 5 and have been included here for completeness. Transcript levels are plotted relative to housekeeping genes (HKGs), and each point represents data from an individual fetus. Black lines denote a generalized linear regression, and grey fill denotes the confidence interval (0.95) around the regression. (TIF 310 kb) [file 12916_2018_1009_MOESM2_ESM.tif]

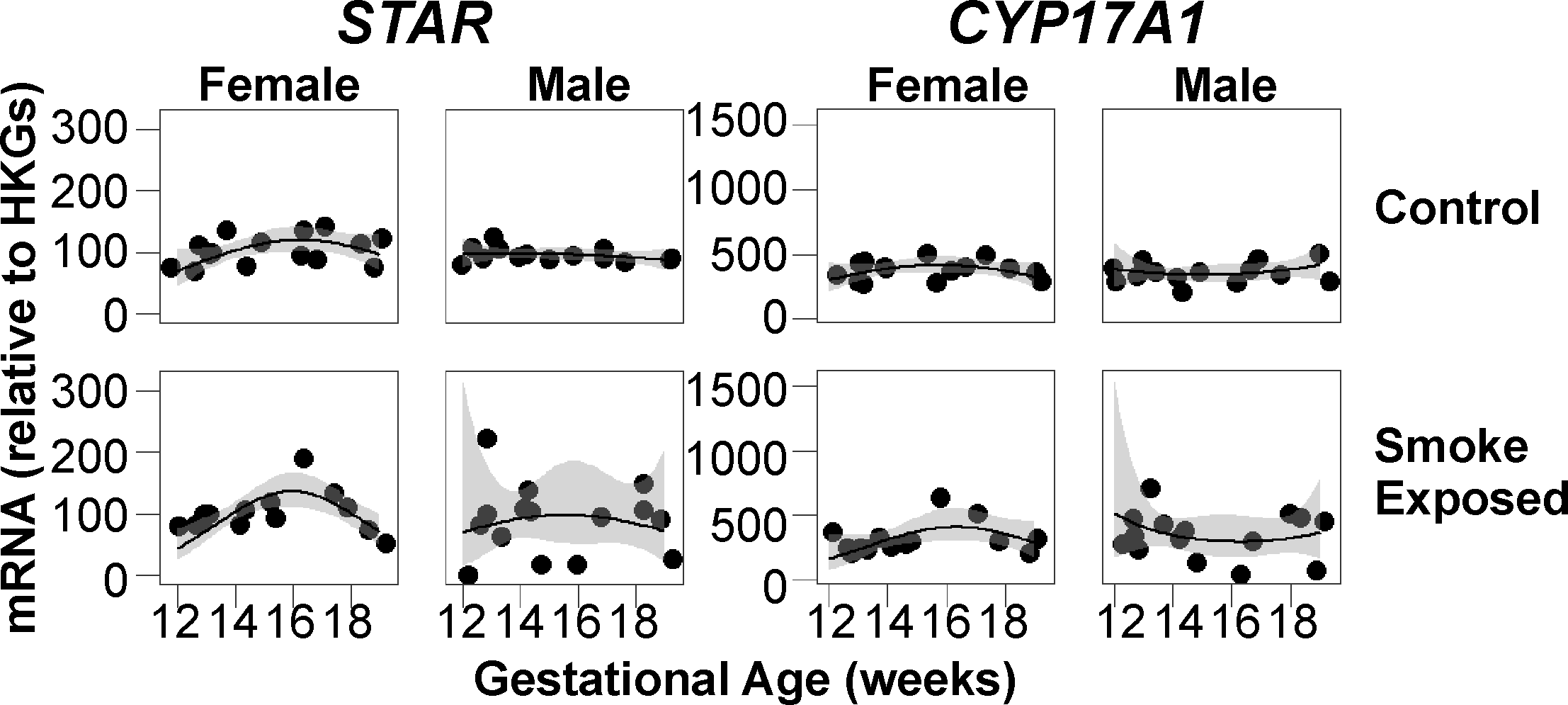

Supplement: Supplementary file 3 — Effect of maternal smoking on STAR and CYP17A1 transcript levels in the fetal adrenal during the second trimester. Maternal smoking was associated with an increase in the variability (Levene’s test) of transcript expression of STAR (P = 0.004) and CYP17A1 (P = 0.02), in male smoke-exposed (SE) fetuses compared to male controls (C). Each point represents data from an individual fetus and transcript levels are expressed relative to HKGs. Black lines denote a generalized linear regression, and grey fill denotes the confidence interval (0.95) around the regression. Data points for females are shown in the left of each panel and males on the right. Data points for controls are shown on the top of each panel and smoke-exposed on the bottom. (TIF 339 kb) [file 12916_2018_1009_MOESM3_ESM.tif]
